# Newborn screening for Duchenne muscular dystrophy: A two‐year pilot study

**DOI:** 10.1002/acn3.51829

**Published:** 2023-06-23

**Authors:** Norma P. Tavakoli, Dorota Gruber, Niki Armstrong, Wendy K. Chung, Breanne Maloney, Sunju Park, Julia Wynn, Carrie Koval‐Burt, Lorraine Verdade, David H. Tegay, Lilian L. Cohen, Natasha Shapiro, Annie Kennedy, Garey Noritz, Emma Ciafaloni, Barry Weinberger, Marty Ellington, Charles Schleien, Regina Spinazzola, Sunil Sood, Amy Brower, Michele Lloyd‐Puryear, Michele Caggana, the Duchenne Muscular Dystrophy Pilot Study Group, Emma Cecilia M. Laureta, Burton L Rochelson, Katherine A. Hogan

**Affiliations:** ^1^ Division of Genetics Wadsworth Center, New York State Department of Health Albany New York USA; ^2^ Department of Biomedical Sciences State University of New York Albany New York USA; ^3^ Department of Pediatrics, Cohen Children's Medical Center Northwell Health New Hyde Park New York USA; ^4^ Departments of Pediatrics and Cardiology Zucker School of Medicine at Hofstra/Northwell Hempstead New York USA; ^5^ Parent Project Muscular Dystrophy Washington DC USA; ^6^ Department of Pediatrics Columbia University Irving Medical Center New York New York USA; ^7^ IQVIA Durham North Carolina USA; ^8^ NewYork‐Presbyterian Cornell New York New York USA; ^9^ NewYork‐Presbyterian Queens Flushing New York USA; ^10^ EveryLife Foundation Washington DC USA; ^11^ Nationwide Children's Hospital Columbus Ohio USA; ^12^ Pediatric Neuromuscular Medicine University of Rochester Rochester New York USA; ^13^ Division of Neonatology, Cohen Children's Medical Center Northwell Health New Hyde Park New York USA; ^14^ Department of Pediatrics Zucker School of Medicine of Medicine at Hofstra/Northwell Hempstead New York USA; ^15^ Department of Pediatrics Lenox Hill Hospital, Northwell Health New York New York USA; ^16^ Division of Neonatal‐Perinatal Medicine at Cohen Children's Hospital/North Shore University Hospital, Northwell Health Manhasset New York USA; ^17^ South Shore University Hospital, Northwell Health Bay Shore New York USA; ^18^ American College of Medical Genetics and Genomics Bethesda Maryland USA; ^19^ Eunice Kennedy Shriver National Institute of Child Health and Human Development National Institutes of Health Bethesda Maryland USA

## Abstract

**Objective:**

Duchenne muscular dystrophy (DMD) is an X‐linked disorder resulting in progressive muscle weakness and atrophy, cardiomyopathy, and in late stages, cardiorespiratory impairment, and death. As treatments for DMD have expanded, a DMD newborn screening (NBS) pilot study was conducted in New York State to evaluate the feasibility and benefit of NBS for DMD and to provide an early pre‐symptomatic diagnosis.

**Methods:**

At participating hospitals, newborns were recruited to the pilot study, and consent was obtained to screen the newborn for DMD. The first‐tier screen measured creatine kinase‐MM (CK‐MM) in dried blood spot specimens submitted for routine NBS. Newborns with elevated CK‐MM were referred for genetic counseling and genetic testing. The latter included deletion/duplication analysis and next‐generation sequencing (NGS) of the *DMD* gene followed by NGS for a panel of neuromuscular conditions if no pathogenic variants were detected in the *DMD* gene.

**Results:**

In the two‐year pilot study, 36,781 newborns were screened with CK‐MM. Forty‐two newborns (25 male and 17 female) were screen positive and referred for genetic testing. Deletions or duplications in the *DMD* gene were detected in four male infants consistent with DMD or Becker muscular dystrophy. One female DMD carrier was identified.

**Interpretation:**

This study demonstrated that the state NBS program infrastructure and screening technologies we used are feasible to perform NBS for DMD. With an increasing number of treatment options, the clinical utility of early identification for affected newborns and their families lends support for NBS for this severe disease.

## Introduction

Duchenne muscular dystrophy (DMD) is the most common pediatric‐onset muscular dystrophy and is characterized by muscle weakness and atrophy, progressive scoliosis, cardiomyopathy, and restrictive lung disease. DMD is an X‐linked disorder with an incidence of approximately 1 in 5000 live male births.^1^, ^2^, ^3^ DMD is part of a spectrum of diseases called dystrophinopathies, caused by pathogenic variants in the *DMD* gene, which codes for the dystrophin protein. Becker muscular dystrophy (BMD), a less severe dystrophinopathy with later onset and slower rate of progression, is typically caused by variants in the *DMD* gene that preserve the open reading frame.^4^, ^5^

Dystrophin is present in skeletal and cardiac muscle where it forms a linkage to the sarcolemma through the dystrophin‐associated protein complex.^6^, ^7^ When muscle fibers are weakened or damaged in DMD or other muscular dystrophies, the enzyme creatine kinase (CK) leaks into the bloodstream. Because levels of serum CK are elevated in people with DMD, CK levels can be used to screen for DMD.^8^, ^9^, ^10^ However, the elevation of CK is an indirect marker of muscle damage and is not specific to DMD or muscular dystrophies. To confirm a diagnosis of DMD, molecular analysis of the *DMD* gene and identification of a pathogenic/likely pathogenic (P/LP) variant are required to provide clinical information for disease prognosis, genetic counseling, and eligibility for mutation‐specific therapies.

Newborn screening (NBS) for DMD has been performed, primarily as pilot studies, in various parts of the world since the 1970s as early detection was believed to be beneficial.^3^, ^11^, ^12^, ^13^, ^14^, ^15^, ^16^, ^17^, ^18^, ^19^, ^20^, ^21^, ^22^ The benefits include early treatments such as physical therapy, allowing families to prepare for supporting a child with DMD by accessing appropriate resources and considering family planning options for future children. Since 2016, the U.S. Food and Drug Administration (FDA) had approved several therapies, including a DMD‐specific corticosteroid and four mutation‐specific antisense oligonucleotides, which mediate exon skipping and allow the production of a smaller but functional dystrophin protein. Currently, 30% of DMD patients have genetic variants that are eligible for approved exon skipping drugs.^23^ Many other treatment options such as gene therapy are under investigation and in clinical trials.^24^, ^25^, ^26^

In December 2019, the FDA authorized a first‐tier screening kit for DMD, which measures the level of creatine kinase‐MM (CK‐MM) in dried blood spots (DBS). Since the CK‐MM isoform is predominantly found in skeletal muscle, it is a more specific marker of skeletal muscle injury than measurement of total CK which measures all isoforms. The FDA‐authorized test is high throughput and provides an effective method for universal DMD screening in newborns.^19^, ^20^, ^21^, ^22^, ^27^, ^28^, ^29^ With the availability of this screen and an increasing number of treatment options, it is probable that DMD meets public health screening criteria for inclusion on the Advisory Committee on Heritable Disorders in Newborns and Children's (ACHDNC) recommended uniform screening panel (RUSP) in the U.S.

Using the FDA‐authorized CK‐MM kit as first‐tier screen, a two‐year consented pilot study to screen newborns for DMD began in October 2019 in New York State (NYS). Because this was a research study, the protocol included investigating the causes of CK‐MM elevation if no P/LP variants were detected in the *DMD* gene. In these cases, additional parental consent was obtained, and genetic analysis of a neuromuscular panel was performed. Data from the first year of the study were previously reported.^30^ In this manuscript, we report on the data from the full two‐year pilot (with some additional follow‐up data on newborns previously reported), provide details of modifications made to improve the study protocol, and make recommendations for implementing NBS for DMD.

## Methods

### Establishing the NYS DMD pilot

The advocacy group, Parent Project Muscular Dystrophy (PPMD), assembled stakeholders with the primary aim of developing additional evidence to support the addition of DMD to the RUSP. Stakeholders included industry partners, healthcare professional groups, advocacy groups, representatives from newborn screening and federal agencies. To gather additional evidence, a two‐year pilot study was performed in NYS to screen newborns at select hospitals. NYS was selected as the location of the pilot study because of the high birth number (over 200,000 per year), its diverse population, and because the NYS NBS Program has extensive experience with performing NBS pilot studies and had the infrastructure in place. The hospital systems (Northwell Health and NewYork‐Presbyterian Hospital) with the highest numbers of births in the state were invited to participate in the pilot study. In addition, a workgroup of clinical experts was formed to identify common data elements (CDEs) for use in the National Institutes of Health Eunice Kennedy Shriver National Institute of Child Health and Human Development's (NICHD) Newborn Screening Translational Research Network (NBSTRN) Longitudinal Pediatric Data Resource (LPDR), operated by the American College of Medical Genetics and Genomics (ACMG), for collecting long term clinical outcome data for diagnosed infants.^30^

The protocol for the pilot study was developed by investigators at the NYS NBS Program and the hospital systems participating in the pilot study. Recruitment materials (brochure and video) and consent forms were developed by the study team to educate parents about the pilot study and translated into Spanish and Chinese by native speaking medical interpreters. The result reports and letters to providers were developed based on the routine reports and letters used by the NYS NBS Program.

The protocol for the pilot and all accompanying materials were approved by the Institutional Review Board (IRB) of the New York State Department of Health as well as those of the participating hospitals.

### Recruitment

Recruitment was performed at the following hospitals: Northwell Health hospitals (Cohen Children's Medical Center, Long Island Jewish Medical Center, North Shore University Hospital, Lenox Hill Hospital, South Shore University Hospital) and NewYork‐Presbyterian (NYP) hospitals (NYP Morgan Stanley Children's Hospital, Allen Pavilion, Weill Cornell Medicine, Lower Manhattan Hospital and NewYork‐Presbyterian Queens). Initially, recruitment involved study staff approaching mothers in person after delivery, describing the pilot study, offering them educational materials including the video and brochure, and then obtaining consent for the newborn to participate in the study. In March 2020, due to the COVID‐19 pandemic, recruitment was transitioned to remote because study staff were not able to make in‐person visits.^31^ At that time, recruitment was performed by telephone or online.^31^ Northwell Health hospitals transitioned to a hybrid approach that included both in‐person and remote recruitment in July 2020, and NYP hospitals followed with hybrid recruitment in August 2020.

At Northwell Health hospitals, any baby whose family could understand English, Spanish, or Chinese was eligible for the study, unless the baby was in the neonatal intensive care unit (NICU) with congenital anomalies or complex medical concerns. At NYP, inclusion criteria were similar except most NICU babies were excluded from the study.^30^

### First tier‐screen

The NYS NBS Program requests a blood specimen be collected via a heel stick from all newborns on a specimen collection card 24–36 h after birth and sent to the NBS program with accompanying mother and infant demographic information. Specimens were shipped overnight at ambient temperature. During accessioning, specimens were manually checked for suitability for testing. Specimens with serum rings or blood clots were considered “suboptimal”; however, they were tested, and an additional specimen was also requested. Specimens collected at <24 h of age were similarly tested, but an additional specimen was requested. In addition, repeat specimens were received from NICU babies based on the NYS NICU protocol requiring the collection of multiple specimens at various timepoints (first specimen at admission to NICU, second specimen between 48 and 72 h and third specimen at discharge or 28 days), and from babies who had borderline results for any other analyte on the NBS panel. Specimens were not tested if the quantity of blood was not considered sufficient for analysis. CK‐MM screening was only performed on specimens from newborns who had been consented to the study.

**Figure 1:**
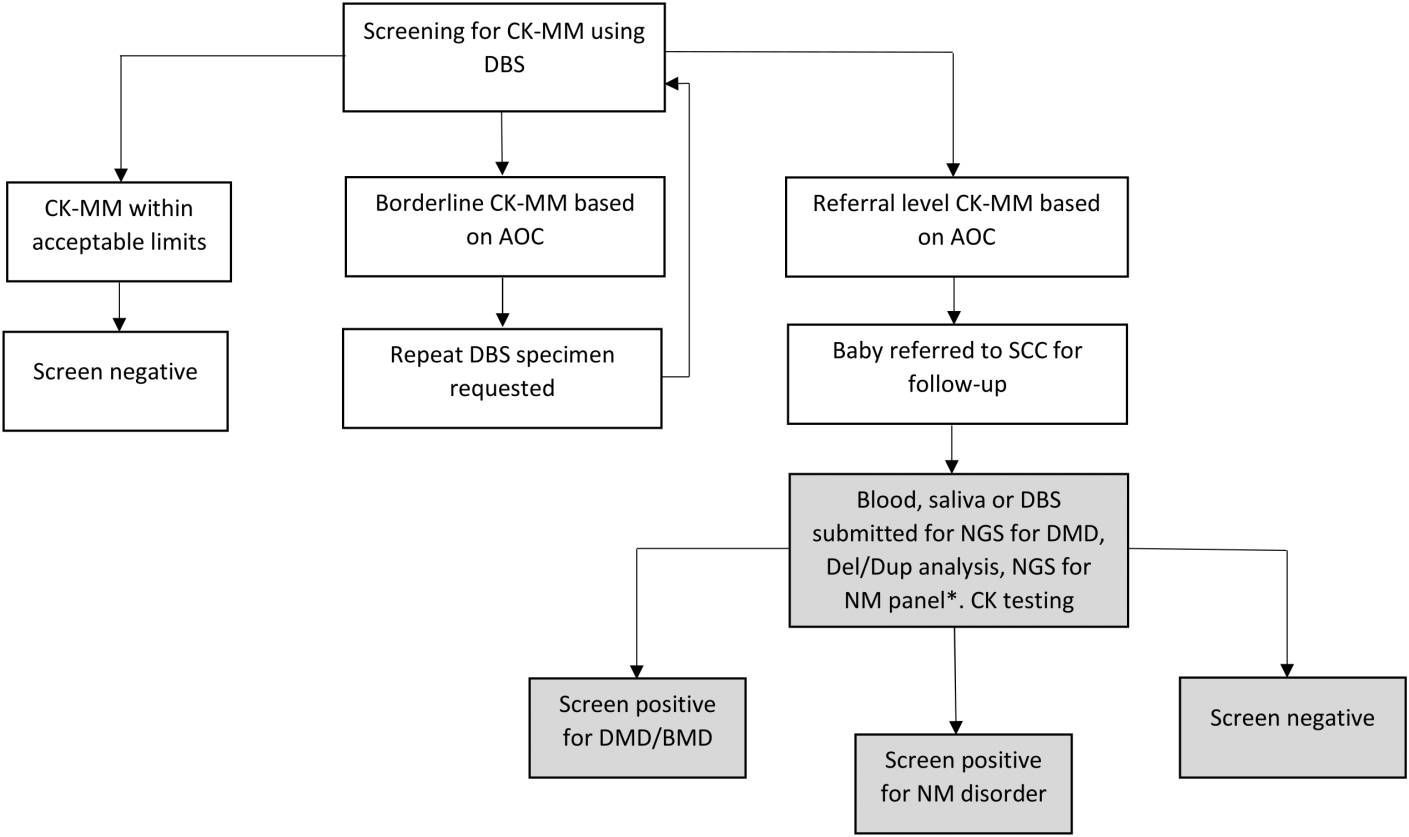
Duchenne muscular dystrophy testing algorithm. CK‐MM, creatine kinase‐MM; DBS, dried blood spot; AOC, age of collection; SCC, specialty care center; NGS, next‐generation sequencing; DMD, Duchenne muscular dystrophy; NM, neuromuscular. *Note that several methods, and gene panels were used based on which laboratory performed the genetic testing (supplementary material). Shading indicates actions performed at specialty care center.

Screening for DMD was performed using the GSP Neonatal Creatine Kinase‐MM kit (PerkinElmer, Waltham, Mass) using the GSP high throughput analyzer as per the manufacturer's instructions. The screening algorithm and results of year one were described previously^30^ (Fig. 1). The cutoffs selected for the assay were based on the age of the newborn at specimen collection (Table 1).^32^ A repeat DBS specimen was requested as soon as possible for repeat CK‐MM screening for any baby with a borderline CK‐MM result.

**Table 1:** Creatine kinase‐MM cutoffs based on age of newborn at specimen collection.

Age at collection (h)	Borderline cutoff (ng/mL)	Referral cutoff (ng/mL)
0–47^1^	≥1990	≥4000
48‐71	≥1430	≥4000
72–167	≥571	≥860
≥168	**–**	≥571

^1^ A repeat specimen is requested for any specimen collected <24 h after birth.

### Genetic counseling and specimen collection for second‐tier screen

Newborns with elevated CK‐MM were referred to a specialty care center with an indication of increased risk of DMD. Initially, in‐person genetic counseling was performed to explain the CK‐MM results to the family. At the specialty care center, consent was obtained for genetic testing, and a blood specimen was collected and submitted for second‐tier testing. Because of the COVID‐19 pandemic, some patients were seen by telehealth and in some instances, families collected and submitted buccal swabs for genetic testing.^31^

Whenever possible, electronic health record, including hospital history and physical, discharge notes, and outpatient clinical notes were reviewed for referred newborns and information such as breech birth, birth trauma and/or birth complication and other clinical findings were recorded.

### Genetic testing

For the pilot study, genetic testing was contracted to a secondary laboratory and was performed free of charge for families whose infants had an elevated CK‐MM. Initially, there were no NYS Clinical Laboratory Evaluation Program (CLEP)‐approved laboratories available that performed molecular analysis for the *DMD* gene using dried blood spot (DBS) specimens and testing was only available on blood or buccal swabs. Approval by CLEP is a regulatory requirement in NYS for testing specimens collected in NYS. Following collection, the specimens were submitted to one of two commercial laboratories for testing. Initially, deletion/duplication analysis and next‐generation sequencing (NGS) of the *DMD* gene were performed. As per the protocol of the pilot study, the specialty care center offered additional testing if no P/LP variants were detected in the *DMD* gene. Depending on which laboratory performed the second‐tier testing, several methods, and gene panels (ranging from 46 to 230 genes) were used by the ordering physician (supplementary material).^30^ Each laboratory used ACMG criteria for variant classification based upon variant evidence at the time of reporting.

During the study, a third commercial laboratory obtained CLEP approval to use DBS for *DMD* NGS. Starting in March 2021, if there was sufficient DBS available after routine NBS was completed, specimens from referred cases were submitted to the approved laboratory for second‐tier testing after consent for genetic analysis was obtained from the family.

## Results

**Figure 2:**
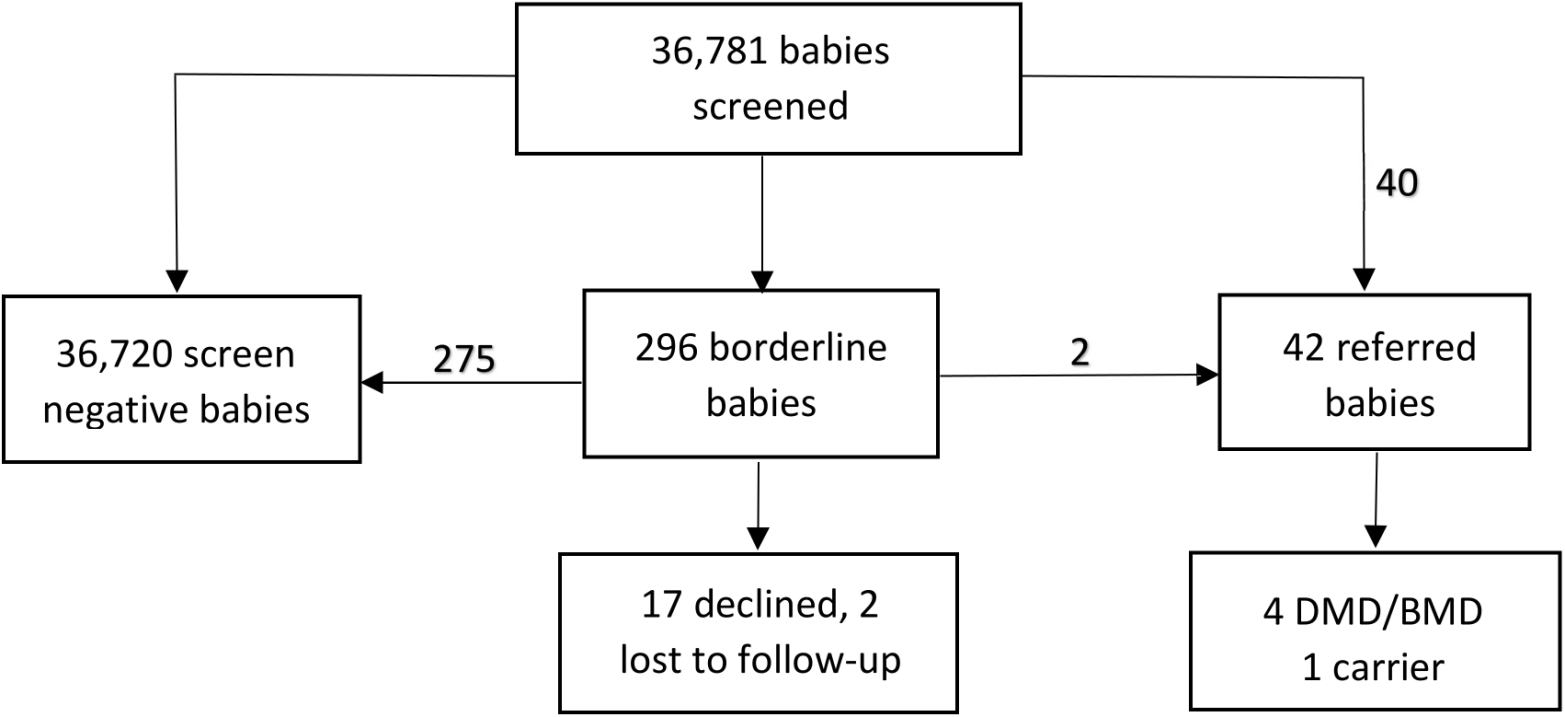
Outcome of screening for Duchenne muscular dystrophy. DMD, Duchenne muscular dystrophy; BMD, Becker muscular dystrophy.

The GSP Neonatal Creatine Kinase‐MM kit underwent extensive validation by the NBS program prior to beginning the pilot study and was approved for use by the NYS CLEP on September 19, 2019. Recruitment for the study began on October 1, 2019 and continued until September 30, 2021. In total, 36,784 babies were recruited to the study with an uptake of 87.1%. A total 36,781 babies were screened using the CK‐MM kit (Fig. 2). Specimens from three consented babies were not tested for CK‐MM: One specimen was lost in transit, and the repeat specimen was sent to a different state for NBS; the second specimen had insufficient quantity for the NBS panel, and a repeat was not received despite multiple requests; and the parents of the third baby withdrew from the study. Characteristics of screened newborns are included in Table 2. The majority of specimens were collected between 24 and 47 h of life. Most of the specimens collected at less than 24 h of age were from NICU babies, and most specimens collected after 48 h of life were repeat specimens collected due to an initial specimen that was unsuitable/suboptimal, collected at less than 24 h of age, had an abnormal or borderline result for an analyte on the NBS panel, or was a repeat specimen collected due to NICU status.

**Table 2:** Demographic information and characteristics of screened newborns.

	Number	%
Number of specimens	39,646	–
Number of unique newborns	36,781	92.8
Number of repeat specimens	2865	7.2
Male	18,654	50.7
Female	17,993	48.9
LBW (<2500 g)	2465	6.7
NBW (2500–3999 g)	32,048	87.1
HBW (≥4000 g)	2264	6.2
In NICU	1681	4.6
Age of collection (h)		
<24	1429	3.6
24–47	35,747	90.2
48–71	793	2.0
72–167	238	0.6
≥168	1437	3.6
QNS (not tested)	8	0.02
Suboptimal (tested)	330	0.8

A repeat specimen was requested for any QNS or suboptimal specimen.

HBW, high birth weight; LBW, low birth weight; NBW, normal birth weight; NICU, neonatal intensive care unit; QNS, quantity not sufficient.

Two hundred and ninety‐six babies (60% male and 40% female) had borderline results, and an additional DBS was requested (Fig. 2). Of 296 babies with borderline results, seventeen families declined to submit a repeat specimen for CK‐MM testing. The DBS CK‐MM concentration of the 17 babies whose parents declined and two babies who were lost to follow‐up ranged from 2018 to 3240 ng/mL. Of the remainder borderline cases, repeat specimens were collected when babies were between 2 and 140 days of age (median: 14 days). Two babies with borderline results were referred based on persistently elevated CK‐MM results from a repeat NBS specimen; all others had a normal CK‐MM on repeat screen using the Neonatal CK‐MM kit.

In total, 42 newborns were referred to a specialty care center for second‐tier testing, and the pediatrician was informed of the abnormal newborn screen results (Fig. 2). A geneticist or genetic counselor contacted the family to explain the NBS results and obtain consent for genetic testing. Descriptive data of the 42 referred cases are presented in Table 3 (newborns with pathogenic/likely pathogenic variants in the *DMD* gene), four (newborns with no P/LP variants detected in the *DMD* gene but who had persistent CK elevation), and Table S1 (newborns who declined *DMD* gene analysis or were lost to follow‐up), Table S2 (newborns whose CK normalized or whose repeat CK is unknown), and Table S3 (newborns who were negative for P/LP variants in the *DMD* gene but were diagnosed with other conditions or were carriers of other conditions). Of the 42 babies who were referred, six families declined genetic testing, and four families declined testing for the neuromuscular panel after no pathogenic variant was detected in the *DMD* gene by sequencing and deletion/duplication analysis (Tables S1 and S2). Babies 20 and 33 were lost to follow‐up, and no genetic testing was performed.

**Table 3:** Characteristics and results of newborns with pathogenic/likely pathogenic variants in the *DMD* gene.

Case ID (sex)	Age at collection (h)	Race/ethnicity	CK‐MM (ng/mL)	DMD gene analysis	Expanded NMD panel^1^	Expanded NMD panel: LP/P	Expanded NMD panel: VUS	Birth events	Clinical history/diagnosis/follow‐up
5 (M)^2^	37	White/non‐Hispanic	7809	15.65 kb duplication of exon 18, predicted out of frame duplication, likely pathogenic	Not completed	N/A	N/A	Delivery C‐section	Molecular diagnosis of Duchenne/Becker muscular dystrophy. Followed in MDA clinic. Last visit at 17 months: Out‐toes but no toe walking. With support can raise self‐off floor. Cannot assess if Gower sign is present. Appears to have trouble raising body with use of one leg. Physical therapy 2x per week and development therapy 1x per week. Mother is DMD carrier. Diagnosis: DMD
6 (M)^2^	24	White/Non‐Hispanic	6384	Deletion of exons 48 and 49, predicted in frame deletion, likely pathogenic	Not completed	N/A	N/A	Delivery C‐section	Followed in MDA clinic. No cognitive or motor delays identified at 7 months. Mother obligate carrier of P variant in *DMD* gene. Maternal family history of atypical adult‐onset BMD. Diagnosis: BMD
22 (M)	331	Hispanic/Dominican Republic	993	Exon 51 deletion. Predicted out of frame, likely pathogenic	Not completed	N/A	N/A	Delivery C‐section, breech	No family history. Diagnosis: DMD/BMD
15 (M)^2^ ^,^ ^3^	27	Asian	18,574	Deletion of exons 3–43, predicted out of frame deletion, likely pathogenic	Invitae 122 gene panel	None identified	*PLEC (*VUSx5), het; *PREPL (*VUS), het	Vacuum‐assisted delivery	Not screened as a newborn. Referred to genetics and enrolled in study because of maternal family history of DMD and markedly elevated CK and Troponin T suggestive of congenital myopathy and hydronephrosis. DMD and gene panel ordered concurrently. Diagnosis: DMD
17 (F)	3360	Other/Hispanic or Latino	1958	*DMD* c.9268G>T (p.Glu3090*) heterozygous, pathogenic	Invitae 109 gene panel	*AGRN* c.3905 C>T p.Thr1302Met heterozygous, pathogenic	*CHRNA1* (VUS), het; *MEGF10* (VUS), het	Delivery C‐section	Mother declined testing for herself and her 4 daughters. Diagnosis: DMD carrier

CK‐MM, creatine kinase‐MM; DMD/BMD, Duchenne/Becker muscular dystrophy; F, female; het, heterozygous; ID, identifier; LP, likely pathogenic; M, male; MDA, Muscular Dystrophy Association; N/A, not applicable; NMD, neuromuscular disease; P, pathogenic; VUS, variant of uncertain significance.

^1^ For molecular methodologies and gene panels see supplementary material.

^2^ Cases 5, 6, and 15 were previously reported.^30^

^3^ Note that baby 15 was a late consent based on family history.

Of the 42 referred babies [25 (60%) males, 17 (40%) females], genetic testing was performed on 34. Four male babies (babies 5, 6, 15, 22) had genetic results consistent with DMD/BMD, and one female infant (baby 17) was a DMD carrier (Table 3). The age of the four confirmed newborns ranged from 41 to 89 days when genetic results were reported. A variant of uncertain significance (VUS) in multiple genes in the NM panel (*COL6L1*, *LAMA2*, *PLEC*, *B4GAT*1) and a 17 kb deletion of uncertain significance in intron 55 and a VUS in the *DMD* gene were detected in a female baby (baby 3) (Table S2). She was also found positive for a heterozygous, likely pathogenic variant in the *RYR1* gene associated with malignant hyperthermia susceptibility or persistent elevation of creatine kinase (the variant was classified as VUS by a second laboratory). A parental segregation study was recommended but not completed. On repeat testing, serum CK activity had normalized and there was no evidence of weakness at 9 months. None of the referred cases was diagnosed with a muscular dystrophy other than DMD/BMD although VUS were detected in genes of the neuromuscular panel for 22 of the 42 babies with elevated CK‐MM and no *DMD* pathogenic variants (Table 4 and Tables S2 and S3). Several babies were either diagnosed with unrelated conditions or were carriers of non‐DMD muscular dystrophies (Table S3). Alagille syndrome was diagnosed as part of clinical care in one newborn (baby 8), unrelated to the elevated CK‐MM detected at NBS. Baby 19 was diagnosed with cerebral palsy and neuromuscular respiratory weakness at 19 months of age. Baby 4 continues to be followed clinically as he had neonatal seizures, hypotonia, speech delay, and neurological complications. Due to concern for inborn error of metabolism in baby 4, complete metabolic workup and exome sequencing with *mitochondrial genome* sequencing and deletion/duplication testing were performed and were all nondiagnostic. Two newborns were carriers of autosomal recessive LAMA2 muscular dystrophy (MD), two newborns were carriers of autosomal recessive limb‐girdle MD, and one newborn was a carrier of MD dystroglycanopathy. All carriers were asymptomatic. At least 13 referred newborns had reported complicated/traumatic birth with nuchal cord complication in three babies, and shoulder dystocia in 10 babies. In addition, vacuum‐assisted vaginal delivery, a tight nuchal cord, hypoxic–ischemic encephalopathy (HIE), and seizures were reported in one newborn (baby 19) and HIE and seizures in a second newborn (baby 23). At least eight of the referred newborns were breech, and at least nine of the referred newborns were admitted to the NICU. CK‐MM normalized in repeat DBS specimens from three referred newborns based on cutoffs for older babies. In 16 newborns, CK activity was measured in serum as requested by the specialty care center by outside laboratories using their reference ranges. Serum CK activity normalized in 12 referred cases. Serum CK activity remained elevated in babies 10 [elevated twofold at 2 years of age], 24 [at 6 months CK activity was 159 units/L (reference range < 136 units/L)], 36 [at 27 weeks CK activity was 324 units/L (reference range 46–171 units/L)], and 39 [at 4 months CK activity was 596 units/L (reference range < 143 units/L)] and only VUS were identified by genetic testing (Table 4). Asymptomatic father of baby 39 also had elevated serum CK activity [1818 units/L (reference range 30–200 units/L)].

**Table 4:** Characteristics and results of referred newborns with no P/LP variants detected in the *DMD* gene but who had persistent CK elevation.

Case ID (sex)	Age at collection (h)	Race/ethnicity	CK‐MM (ng/mL)	DMD gene analysis	Expanded NMD panel^1^	Expanded NMD panel: LP/P	Expanded NMD panel: VUS	Birth events	Clinical history/diagnosis/follow‐up
10 (F)^2^	25	Declined	5365	Negative	EGL	None identified	*LAMA2* (VUS), het	Unknown	Elevated CK at 2 years. Gross motor delays. Referred to MDA Clinic
39 (F)	24	Other/ Hispanic or Latino	6127	Negative	PEG	None identified	*CAV3* (VUS), het; *LAMA2* (VUS), het; *NEB* (VUS), het; *PLEC* (VUS), het; *CHRND* (VUS), het; *DYNC1H1* (VUS), het; *MTM1* (VUS), het; *SMCHD1* (VUS), het; *SCN4A* (VUS), het	C‐section due to failed induction	Persistent CK elevation; followed at NM clinic; CK elevated at 500–600 at 4 months of age (RR <143 U/L). Development was normal at 12 months of age. Asymptomatic father's CK also elevated (1818 U/L) (RR: 30–200 U/L). His genetic testing is pending
36 (M)	26	White/ Non‐Hispanic	5518	Negative	PEG	None identified	*COL6A1* (VUS), het	Loose nuchal cord	CK‐MM normalized at 9 days in DBS. However, CK elevated at 27 weeks in serum
24 (M)	26	Declined	17,565	Negative	EGL and PEG 90 gene panel	None identified	*COL6A1 (VUS)*, het; *TTN* (VUS), het; *UBA1* (VUS), het	C‐section due to prolonged labor	CK elevated at 6 months. Neurological examination benign with normal development at 11 months

CK‐MM, creatine kinase‐MM; DBS, dried blood spot; DMD Duchenne muscular dystrophy; EGL, Emory Genetics Laboratory; F, female; het, heterozygous; ID, identifier; LP, likely pathogenic; M, male; MDA, Muscular Dystrophy Association; NM, neuromuscular; NMD, neuromuscular disease; P, pathogenic; PEG, Perkin Elmer Genomics; RR, reference range; VUS, variant of uncertain significance.

^1^ For molecular methodologies and gene panels see supplementary material.

^2^ Case 10 has previously been reported.^30^

Of the four DMD positive babies, baby 22 was a 3310 g (gestation: 38 weeks, 0 days) male dizygotic twin whose initial specimen was collected at 29 h and had a CK‐MM of 3592 ng/mL (NR < 1990 ng/mL). This result was considered borderline, and a repeat specimen was requested. The repeat specimen was collected at 14 days, and the CK‐MM was 993 ng/mL (NR < 571 ng/mL). Molecular analysis detected a 1.15 kb deletion in exon 51 of the *DMD* gene resulting in an out‐of‐frame deletion consistent with DMD/BMD. Twin A was a 2950 g male with CK‐MM of 413 ng/mL (NR < 1990 ng/mL) at 29 h. There was no family history of DMD. Genetic testing of the mother and twin A indicated no P/LP variants in the *DMD* gene. Follow‐up examination of baby 22 at 5 months indicated decreased strength and coordination. Early intervention and physical therapy were initiated to assist in slowing disease progression and preserve muscle function for as long as possible.

The second confirmed DMD baby, baby 6, had a deletion of exons 48–49 in the *DMD* gene and has a symptomatic grandfather with the same deletion. At 21 months, the infant had no significant motor or cognitive delays but is predicted to have a BMD phenotype based upon the family history and in‐frame variant.

The other two babies with confirmed P/LP variants in the *DMD* gene, babies 5 and 15, also had a maternal family history of a dystrophinopathy. The mother of male baby 15 was a known DMD carrier with a family history of DMD. This baby had an extremely high CK‐MM at birth (18,574 ng/mL), and molecular analysis indicated an out‐of‐frame deletion of exons 3–43 confirming DMD. At 2 years old, he appeared to have some weakness, mainly in lower extremities, speech delay and suspected comorbidity with autism spectrum disorders. Baby 5 had a predicted out‐of‐frame duplication in exon 18. At 17 months of age, the infant had some muscle weakness and is participating in physical and developmental therapy.

None of the four babies with a DMD/BMD diagnosis were eligible for FDA‐approved exon skipping drugs and currently none are on medication specific to DMD. However, they will be eligible for clinical trials, including those for steroids and perhaps future trials of molecular treatments.

Baby 17, the confirmed carrier, was a female with an initial borderline result (CK‐MM: 3885 ng/mL at 28 h of age, NR < 1990 ng/mL) who was subsequently referred based on a specimen collected at 140 days (CK‐MM: 1958 ng/mL; NR < 571 ng/mL). This baby had a stop codon in exon 63 [c.9268G>T (p.Glu3090*)] of the *DMD* gene and was confirmed as a carrier. There was no known family history of DMD/BMD for baby 17. Follow‐up examination at 6 months was unremarkable.

The mother of a female baby (data not shown) with a borderline result (CK‐MM: 3101 ng/mL, NR < 1990 ng/mL) was a known DMD carrier. Although the parents declined molecular testing, based on the elevated CK‐MM, the expectation is that their female newborn is also a carrier.

Based on the referral and borderline level CK‐MM cutoffs used in this pilot and confirmation of five newborns with pathogenic variants in the *DMD* gene, the false positive rate for the CK‐MM component of the study was 0.9% (323/36,768) (1.0% for males and 0.7% for females). The positive predictive value (PPV) for the CK‐MM screen for DMD/BMD was 14.7% (5/34) (19% for males and 7.7% for females). The recall rate, inclusive of borderline and referred newborns, was 0.9% (336/36,781) (1.1% for males and 0.7% for females).

## Discussion

Because muscle damage in DMD/BMD is irreversible, it is important to identify DMD/BMD patients as early as possible for treatment. Early diagnosis and molecular characterization of newborns with DMD will enable mutation‐specific treatment when available and the opportunity to participate in clinical trials for novel therapies. While not covered under Wilson and Jungner screening criteria, additional value to the information gained by DMD/BMD newborn screening would be to identify family members at risk who would not otherwise be identified and allow families to access supportive services and make informed decisions regarding family planning. Furthermore, since female carriers of a pathogenic variant in the *DMD* gene can develop symptoms of dystrophinopathy ranging from mild muscle weakness to significant disability similar to BMD, NBS would also benefit females. Female carriers also have a higher risk of developing cardiomyopathy and would benefit from appropriate recommendations including regular cardiac evaluations.^33^ The identification of a female carrier in the NYS NBS pilot led to the development of an algorithm for evaluation of screen positive females and family members.^34^

NBS pilot studies are crucial for understanding whether performing large‐scale screening for a specific disorder is feasible and beneficial, and for identifying challenges that require resolution prior to full implementation. These were the reasons for including testing for a neuromuscular panel as part of this pilot study as one of the aims was to identify causes other than DMD for CK‐MM elevation in newborns. Additionally, this approach replicated the current standard of care approach to the persistent elevation of CK.

After screening 36,781 newborns, the NYS pilot identified three male newborns with DMD/BMD based on molecular analysis of the *DMD* gene (Table 3). The pilot also confirmed DMD in baby 15 who was included in the study as a late consent due to family history of DMD. Excluding this baby, the incidence of DMD/BMD was 1 in 6218 males (95% CI 1 in 1098 to 1 in 35,224), which is similar to that previously reported.^1^, ^2^, ^3^

The CK‐MM screen detected a confirmed female DMD carrier and flagged a second possible carrier, but the screen is not expected to detect all female carriers because only 50–70% of carriers have elevated serum CK.^35^, ^36^ Furthermore, not all BMD cases will be identified because there may not be sufficient muscle damage to cause an increase in CK‐MM at the time of NBS^28^. Disease progression in BMD can be variable with severe BMD cases more likely to have muscle damage and childhood onset. More severe BMD cases could potentially be more likely to be detected by NBS.

Creatine kinase levels can be elevated transiently due to muscle trauma.^37^ Trauma at birth leads to elevated CK results.^38^ Amato et al. reported markedly elevated levels of CK‐MM following vaginal delivery especially if complicated by forceps, vacuum, and breech presentation.^39^ In the NYS study, several referred newborns had shoulder dystocia, shoulder dislocation, or were born by breech delivery. Shoulder dystocia was reported in 24% of referred newborns as compared to approximately 1–10% in the general population.^40^, ^41^ With a first‐tier CK‐MM assay, traumatic births could lead to false positive results. If a repeat specimen is collected at a distant time from birth and the CK result falls within the normal range, the expectation would be that the initial elevated result was a false positive. Mendell et al. attributed early CK elevation in DBS of newborns whose CK normalized and who tested negative for DMD pathogenic variants to birth trauma.^3^ In cases of traumatic birth, repeat CK screening at a later time point (e.g., 1‐month well‐baby visit) should be considered to determine whether CK elevation is transient or whether the level remains elevated indicating an increased risk of DMD or another muscular dystrophy for which follow‐up would be advised.

NBS pilot studies are staff‐intensive and costly. Testing a subset of specimens for a separate disorder is tedious and complicated. Great care is needed to ensure testing is only completed on babies whose parents consented. In the case of the NYS pilot, CLEP approval was required for testing. Because genetic testing of the *DMD* gene from DBS was not CLEP‐approved at the beginning of the study, a second specimen had to be collected for genetic testing. This step was a barrier for many parents and contributed to delays in reporting results especially during the COVID‐19 pandemic which was associated with multiple challenges.^42^ Recruitment numbers fell during the initial months of the pandemic and there were delays in submission of repeat specimens, scheduling genetic counseling appointments and delays in clinical evaluation of referred newborns. In some cases, these delays may have contributed to families declining testing. Remote recruitment, telehealth visits, and remote sample collection assisted in overcoming some of these unavoidable challenges.^31^

There are other limitations to this study. Although the CK‐MM screen is an effective method for detecting DMD in newborns, it flags any newborn with elevated CK‐MM due to reasons unrelated to the target condition. This could include newborns with traumatic births or newborns with other muscular dystrophies.^19^, ^28^, ^43^ It is important to perform follow‐up of newborns with persistently elevated CK‐MM to understand the reason for elevation. For example, of the newborns who were referred in our study and had VUS in the neuromuscular panel, it is unclear whether CK‐MM was elevated due to the effect of these variants or other factors. These VUS do not establish a diagnosis and should not be used for family member screening at this time. The classification of variants may change over time due to new variant interpretation guidelines and/or new information. If a VUS is reclassified, the laboratories will update the report with the new interpretation and provide notification to the ordering providers who will then report reclassifications to patients/parents. Based on the results of this pilot study, notably the detection of a considerable number of VUS in non‐*DMD* genes, we recommend that performing the NGS of the NM panel be part of the follow‐up performed by the specialty care center, if CK activity or CK‐MM concentration remains elevated or the baby exhibits symptoms. Another study limitation is that some families declined further testing; therefore, follow‐up data are not available. Additionally, because symptoms of DMD do not manifest until 2–3 years of age, we are not yet aware of any false‐negative results. The establishment of comprehensive registries will assist in identification of false‐negative cases.

**Figure 3:**
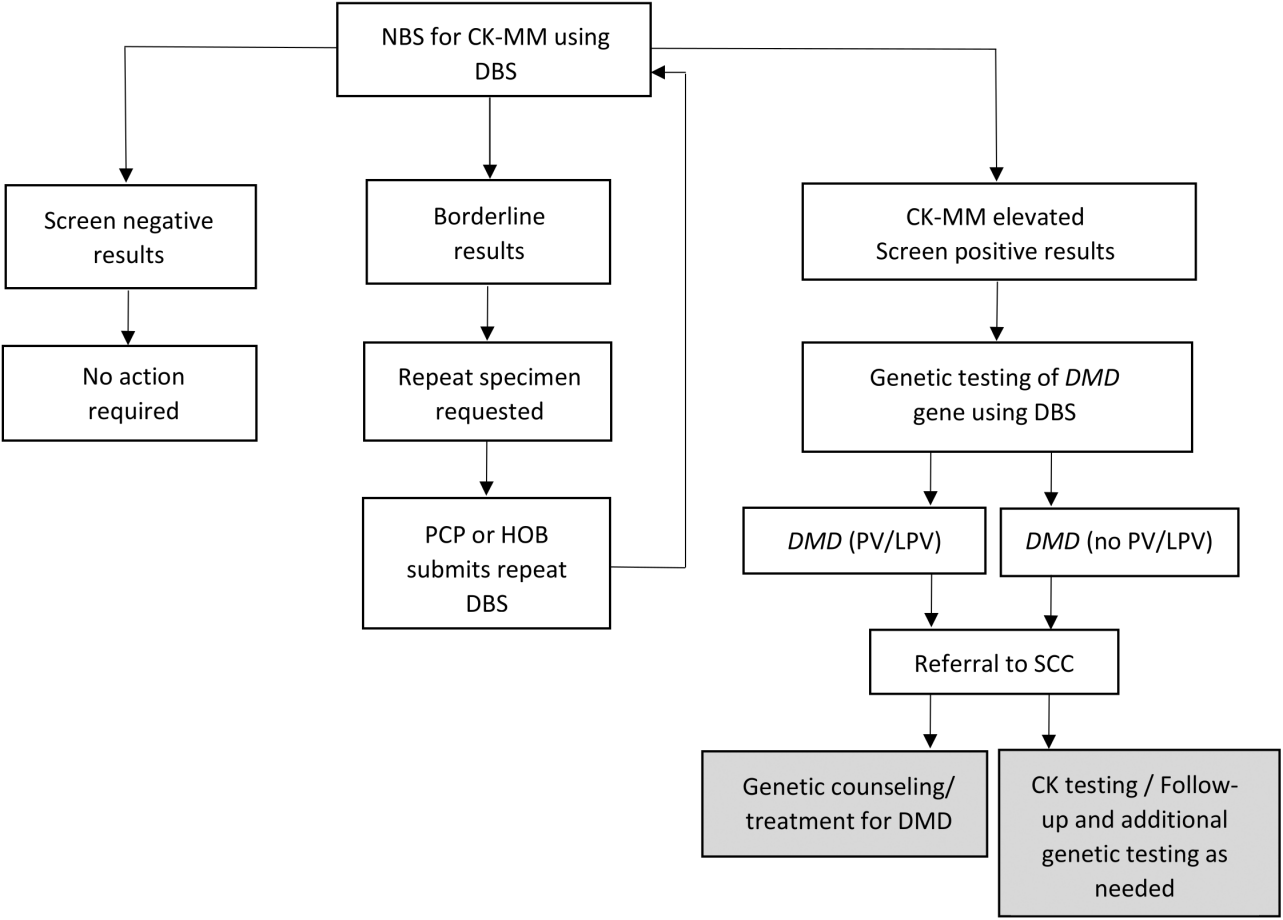
Potential Duchenne muscular dystrophy screening algorithm. NBS, newborn screening; CK‐MM, creatine kinase‐MM; DBS, dried blood spot; DMD, Duchenne muscular dystrophy; PCP, primary care physician; HOB, hospital of birth; PV/LPV, pathogenic variant/likely pathogenic variant; SCC, specialty care center. Shading indicates actions performed at specialty care center.

For future DMD screening, we propose reflex testing from first‐tier to second‐tier screening, similar to the process many NBS programs follow for cystic fibrosis (CF) (Fig. 3). The typical algorithm for CF is to measure the level of the analyte immunoreactive trypsinogen and if elevated, to perform molecular analysis using DBS specimens.^44^ NGS technology is increasingly used to identify *CFTR* variants.^45^, ^46^ P/LP variants are then reported to the pediatrician and a specialty care center and diagnostic testing (sweat test) is performed to confirm CF.^47^ A similar approach would involve an initial measurement of CK‐MM in DBS and if elevated, molecular analysis of the *DMD* gene using DBS specimens.^20^ Education and genetic counseling would be given to those with P/LP variants at follow‐up for DMD. This protocol would eliminate the need to obtain consent for genetic testing and decrease the turn‐around time for obtaining results. If the NYS pilot had only referred newborns with P/LP variants and VUS in the *DMD* gene, then only six newborns would have been referred (Babies 3, 5, 6, 15, 17, 22) for follow‐up and the PPV for the screening would have been 83%. The caveat for this algorithm is that some NBS Programs may not have the capability of performing molecular testing of the *DMD* gene and may require the use of a reference laboratory.

To prevent false‐negative results, sequential CK‐MM testing should be assessed for premature/low birth weight (LBW) newborns whose CK‐MM values are low at birth. This recommendation is similar to that for premature/LBW/NICU babies for screening for other disorders on the NBS panel.^48^ Additionally, with the reduction in costs of molecular testing, specimens with borderline results could also undergo molecular testing to reduce the recall rate. The NBS program would then report elevated CK‐MM results and *DMD* genetic results (including VUS) on the routine NBS report. The specialty care center would then determine follow‐up testing, including measurement of CK activity and additional genetic testing if warranted and desired by the parents. This would be part of the routine standard of care provided by the specialty care center. If CK‐MM normalizes and the child remains symptom‐free, follow‐up can be discontinued. Further follow‐up testing might be warranted if CK‐MM concentration remains elevated and/or the child has symptoms. Relaying this information to the NBS program would allow for completion of short‐term follow‐up by the NBS program.

An alternative course of action for all newborns with elevated CK‐MM would be to repeat the CK‐MM at a later time point (e.g., at 1 month of age) and if it remains elevated, then reflex testing to second‐tier could be performed. This option may increase recalls, but fewer babies would require second‐tier testing and follow‐up at specialty care centers as CK‐MM would have stabilized in the majority of the babies.

In this pilot, three babies with confirmed DMD who had specimens collected between 24 and 37 h had CK‐MM levels above 6300 ng/mL (referral cutoff ≥4000 ng/mL) and one newborn with confirmed DMD whose specimen was collected at 14 days had a CK‐MM value of 992.7 ng/mL (referral cutoff ≥571 ng/mL). Based on this limited number of confirmed cases, the referral cutoff for specimens collected between 0 and 47 h of age could potentially be raised to 5000 ng/mL resulting in 18 fewer referrals and no missed DMD cases. Furthermore, if the borderline cutoff for the 0–47‐h old category was raised from ≥1990 to ≥3000 ng/mL, it would have reduced the need for repeat DBS requests by 82% and not missed either of the DMD cases with initial borderline results. Raising the threshold could potentially result in missing female carriers but may be justified if the goal of NBS is to detect DMD cases. Additional screening data are required to determine whether fine‐tuning the cutoff values can improve test parameters. We are currently analyzing pilot data with respect to factors that influence CK‐MM levels in order to optimize assay cutoff values. For example, our data and previous reports indicate that LBW/premature babies have lower CK‐MM values than normal birth weight newborns.^21^, ^22^ Therefore, lower cutoffs may be implemented for this category of newborns.^21^ Alternatively, CK‐MM screening at a later time point should be considered as previously recommended.^28^ In our pilot study, because our NICU protocol requires submission of multiple specimens, we performed screening at later time points for NICU babies, many of whom were LBW/premature.

In conclusion, although none of the DMD‐affected newborns detected through this pilot study have yet started medical treatment, they benefited from early diagnosis through early genetic counseling, early intervention and physical therapy and could potentially participate in clinical trials. The pilot study allowed us to gather evidence to support the nomination of DMD to the RUSP and to establish a path forward for NBS for DMD. In addition, clinical outcome data will be collected on diagnosed newborns and be available through the NBSTRN's LPDR.

## Author Contributions

N.P.T., D.G., N.A., W.K.C., A.K., G.N., E.C., A.B., M.L‐P., and M.C. were involved in conception and design of the study. N.P.T., D.G., W.K.C., B.M., S.P., J.W., C.K‐B., L.V., D.H.T., L.L.C., N.S., B.W., M.E., C.S., R.S., S.S., E.C.M.L., B.L.R., and K.A.H. were involved in acquisition and analysis of data. N.P.T., D.G., N.A., W.K.C, and M.L‐P. were involved in drafting a significant portion of the manuscript.

## Conflicts of Interest

W.K.C. is on the Board of Directors of Prime Medicine. The funders had no role in the design of the study; in the collection, analyses, or interpretation of data; in the writing of the manuscript; or in the decision to publish the results. The authors declare no conflict of interest.

## Supporting information


Table S1.

Table S2.

Table S3.

File S1.
