# Supplementary material for: Newborn screening for Duchenne muscular dystrophy: A two‐year pilot study
Source: Ann Clin Transl Neurol. 2023 Jun 23;10(8):1383–96. doi: 10.1002/acn3.51829 (PMC10424650; doi:10.1002/acn3.51829)
Supplement: Supplementary file 1 — Table S1. [file ACN3-10-1383-s001.docx]

Supplementary Table 1. Characteristics and results of referred newborns who declined *DMD* gene analysis or were lost to follow-up

| **Case ID (Sex)** | **Age at Collection (hour)** | **Race/ Ethnicity** | | **CK-MM (ng/ml)** | **Birth Events** | **Clinical History / Diagnosis / Follow-up** |
| --- | --- | --- | --- | --- | --- | --- |
| ^‡^1 (M) | 31 | Black or African American/Non-Hispanic | 4,593 | | Breech | Normalized CK at 2 months, no evidence of weakness. Normal development at 6 months |
| 23 (M) | 6 | Asian | 6,112 | | C-section for breech presentation, HIE, seizures | Normalized CK-MM at 68 hours and 223 hours. Normal development at 5 months. Parents declined genetic testing |
| ^‡^7 (F) | 24 | White | 5,054 | | Unknown | Parents report normal development at 7 months. Declined follow-up |
| 28 (F) | 32 | White/ Hispanic or Latino | 4,025 | | Born with poor tone and color with tight nuchal cord | Normal development at 18 months. Speech delay led to referral for early intervention |
| ^‡^2 (F) | 25 | Asian | 8,399 | | Unknown | Declined follow-up |
| 20 (F) | 24 | Declined | 7,648 | | Shoulder dystocia, breech | Lost to follow-up |
| 33 (M) | 24 | Declined | 4,698 | | Shoulder dystocia, fractured clavicle | Lost to follow-up |

Case 19 (presented in supplementary Table 3) also declined genetic analysis. ^‡^Cases 1, 2 and 7 were previously reported.^30^ ID, identifier; CK-MM, creatine kinase-MM; M, male; F, female; HIE, hypoxic ischemic encephalopathy.
