# Supplementary material for: Newborn screening for Duchenne muscular dystrophy: A two‐year pilot study
Source: Ann Clin Transl Neurol. 2023 Jun 23;10(8):1383–96. doi: 10.1002/acn3.51829 (PMC10424650; doi:10.1002/acn3.51829)
Supplement: Supplementary file 2 — Table S2. [file ACN3-10-1383-s004.docx]

Supplementary Table 2. Characteristics and results of referred newborns whose CK normalized or whose repeat CK is unknown

| **Case ID (Sex)** | **Age at Collection (hour)** | **Race/ Ethnicity** | | **CK-MM (ng/ml)** | **DMD Gene Analysis** | **Expanded NMD Panel**** | **Expanded NMD Panel: LP/P** | **Expanded NMD Panel: VUS** | **Birth Events** | **Clinical History / Diagnosis / Follow-up** |
| --- | --- | --- | --- | --- | --- | --- | --- | --- | --- | --- |
| ^‡^3 (F) | 24 | Asian | 4,895 | | No pathogenic variants | EGL | *RYR1*  heterozygous, likely pathogenic (variant identified by another lab as VUS | *DMD (VUS:* 17kb deletion of intron 55), het | Normal vaginal delivery | Normalized CK and no evidence of weakness at 9 months |
| ^‡^11 (M) | 24 | White/ Non-Hispanic | 4,154 | | Negative | EGL | None identified | *TTN (*VUS), het | Normal vaginal delivery | Normalized CK and no evidence of weakness at 1 month |
| ^‡^16 (F) | 25 | Declined | 4,507 | | Negative | EGL | None identified | *RYR2* (VUS), het; *TTN* (VUS), het | Unknown | Normalized CK at 9 days. Declined further follow up |
| 18 (F) | 24 | White/ Non-Hispanic | 4,611 | | Negative | Invitae 143 gene panel | None identified | *CAV3* (VUS), het *NEB* (VUS), het *GAA* (benign pseudodeficiency allele), het | C-section | Normalized CK, no evidence of weakness |
| 26 (M) | 24 | White/ Non-Hispanic | 4,321 | | Negative | Invitae 143 gene panel | None identified | *COL12A1* (VUS), het; *STIM1* (VUS), het; *AMPD1* (VUS), het; *MAP3K20 (VUS), het; MEGF10* (VUS), het | Shoulder dystocia | Normalized CK at 14 days |
| 30 (M) | 24 | White/ Non-Hispanic | 6,566 | | Negative | Invitae 131 gene panel | None identified | *GAA* (benign pseudodeficiency allele), het; *ORAI1* (VUS), het | Vacuum assisted vaginal delivery for shoulder dystocia | Normalized CK |
| 34 (F) | 29 | White/ Non-Hispanic | 4,164 | | Negative | Invitae 143 gene panel | None identified | None identified | Nuchal cord complication | Normalized CK-MM at 10 days |
| 35 (M) | 25 | White/ Asian | 6,107 | | Negative | Declined | N/A | N/A | Unknown | Normalized CK at 11 weeks |
| 42 (M) | 29 | African American/Non-Hispanic | 11,997 | | Negative | Invitae 230 gene panel | None identified | *ITGA7* (VUS),het; *KLHL41* (VUS), het; *LAMB2* (VUS), het; *OPA1* (VUS), het; *PNPLA8* (VUS), het | C-section. Required deep suctioning of heavy meconium & CPAP | Normalized CK at 2 months of age, no evidence of weakness |
| 37 (F) | 25 | White/ Non-Hispanic | 4,298 | | Negative | Declined | N/A | N/A | Shoulder dystocia | Normal physical exam; Elevated CK-MM of unknown etiology |
| ^‡^13 (M) | 29 | African American | 5,128 | | Negative | EGL | None identified | *SIL1* (VUS), het | Unknown | CK was not repeated. Declined further follow up |
| ^‡^14 (F) | 24 | Native Hawaiian or Other Pacific Islander/Hispanic or Latino | 12,002 | | Negative | Invitae 143 gene panel | None identified | *AMPD1* (VUS), het | Normal vaginal delivery | CK was not repeated. Family moved out of NYS |
| 21 (M) | 24 | White/ Non-Hispanic | 4,849 | | Negative | Invitae 143 gene panel | None identified | *COL6A3* (VUS), het; *PLEC* (VUS), het; *PYROXD1* (VUS), het | Vacuum assisted delivery for shoulder dystocia | Elevated CK-MM of unknown etiology; prenatally detected ventricular septal defect |
| 41 (M) | 24 | White/ Non-Hispanic | 5,177 | | Negative | PEG | None identified | *ITGA7* (VUS), het *NEB* (VUS), het *CHRNE* (VUS), het | Normal vaginal delivery | No repeat CK available; gene testing results essentially unremarkable |
| 38 (F) | 24 | White/ Non-Hispanic | 4,407 | | Negative | Insufficient specimen/ Declined further testing | N/A | N/A | C-section | Expanded panel not performed due to insufficient specimen. Declined further testing |
| 29 (M) | 26 | White/ Non-Hispanic | 7,310 | | Negative | Declined | N/A | N/A | Breech | Elevated CK-MM of unknown etiology |

**For molecular methodologies and gene panels see supplementary material. ^‡^Cases 3, 11, 13, 14 and 16 have previously been reported.^30^ ID, identifier; CK-MM, creatine kinase-MM; DMD, Duchenne muscular dystrophy; NMD, neuromuscular disease; het, heterozygous; LP, likely pathogenic; P, pathogenic; VUS, variant of uncertain significance; M, male; F, female; EGL; Emory Genetics Laboratory; PEG, Perkin Elmer Genomics; N/A, not applicable; CPAP, continuous positive airway pressure.
