# Supplementary material for: Newborn screening for Duchenne muscular dystrophy: A two‐year pilot study
Source: Ann Clin Transl Neurol. 2023 Jun 23;10(8):1383–96. doi: 10.1002/acn3.51829 (PMC10424650; doi:10.1002/acn3.51829)
Supplement: Supplementary file 3 — Table S3. [file ACN3-10-1383-s003.docx]

Supplementary Table 3. Characteristics and results of referred newborns who were negative for P/LP variants in the *DMD* gene but were diagnosed with other conditions or were carriers of other conditions

| **Case ID (Sex)** | **Age at Collection (hour)** | **Race/ Ethnicity** | | **CK-MM (ng/ml)** | **DMD Gene Analysis** | **Expanded NMD Panel**** | **Expanded NMD Panel: LP/P** | **Expanded NMD Panel: VUS** | **Birth Events** | **Clinical History / Diagnosis / Follow-up** |
| --- | --- | --- | --- | --- | --- | --- | --- | --- | --- | --- |
| ^‡^8 (F) | 27 | Asian | 4,370 | | Negative | EGL | None identified | None identified | Breech | CK normalized at 4 months. Baby had a congenital heart defect and very high liver enzyme. Clinical genetics evaluation revealed *JAG1* pathogenic variant consistent with Alagille syndrome |
| 40 (M) | 1 29 | Hispanic | 5,198 16,204 | | Negative | PEG | *LAMA2* exon 41 - c.5914C>T (p.Gln1972Ter) heterozygous, pathogenic | *GNE* (VUS), het | Shoulder dystocia, respiratory distress syndrome | Carrier of LAMA2. Elevated CK-MM of unknown etiology |
| ^‡^9 (F) | 24 | Asian | 6,007 | | Negative | EGL | *GNE* c.218G>A, heterozygous, pathogenic, associated with Sialuria (AD) Nonaka myopathy (AR) | *POMT1 (*VUS), het; *TTN (*VUSx3), het | Breech | CK not repeated. Clinical evaluation at 1 month showed no evidence of Sialuria |
| 12 (F) | 24 | White/ Non-Hispanic | 4,150 | | Negative | EGL | *SGCA,* heterozygous, pathogenic, no second variant identified | *TTN (*VUS), het | Vacuum assisted delivery for shoulder dystocia | Normalized CK, no evidence of muscle weakness at 1 month. Normal development at 2 years. Family received genetic counseling regarding AR inheritance of limb girdle muscular dystrophy and offered parental testing |
| 25 (M) | 25 | Declined | 4,890 | | Negative | PEG | *PLEC* c.11974G>T (p.Glu3992Ter) heterozygous, likely pathogenic. Associated disease: Muscular dystrophy, limb girdle, autosomal recessive 17; *PLEC* related epidermolysis bullosa simplex. | *LAMA2*(VUS), het; *LMNA* (VUS), het | Shoulder dystocia | Elevated CK-MM of unknown etiology. Carrier of Limb girdle muscular dystrophy |
| 27 (M) | 26 | White/ Non-Hispanic | 4,310 | | Negative | PEG | *PLEC* c.11974G>T (p.Glu3992Ter) heterozygous, likely pathogenic. Associated disease: Muscular dystrophy, limb-girdle, autosomal recessive; *PLEC* related epidermolysis bullosa simplex. | *NEB* (VUS), het; *RYR1* gene (VUS), het | Delivery C-section, breech | Elevated CK-MM of unknown etiology. Carrier of Limb girdle muscular dystrophy |
| 31 (M) | 24 | Declined | 5,317 | | Negative | Invitae 143 gene panel | *LAMA2* heterozygous, pathogenic c.7377dup (p.Leu2460Serfs*2) | None identified | Vacuum assisted vaginal delivery | Carrier of autosomal recessive LAMA2 muscular dystrophy |
| 32 (M) | 24 | White/ Hispanic | 4,870 | | Negative | PEG | *POMT1* c.692T>A, (p.Leu231Ter) heterozygous likely pathogenic | *DYNC1H1* (VUS), het; *PMP22* (VUS), het; *COL6A2* (VUS) het; *ITGA7* (VUS), het | Shoulder dystocia | Carrier of Muscular dystrophy- dystroglycanopathy. Elevated CK-MM of unknown etiology |
| ^‡^4 (M) | 58 | White/ Non-Hispanic | 4,850 | | Negative | EGL | None identified | *DYSF* (VUS), het *PLEC* (VUS), het *RYR2* (VUS), het | Vaginal delivery. Seizures, respiratory distress, HIE | Normalized CK at 10 days. At birth there was concern for inborn error of metabolism because of standard newborn screening panel. Complete metabolic workup and whole exome sequencing with mitochondrial genome seq/del & dup were negative |
| 19 (M) | 32 | White/ Non-Hispanic | 4,308 | | Not completed | Not completed | N/A | N/A | Vacuum assisted delivery, tight nuchal cord, HIE, seizures | Normalized CK-MM at 25 days and 45 days. At 19 months diagnosed with cerebral palsy, restrictive lung disease, neuromuscular respiratory weakness, dysphagia, sialorrhea |

**For molecular methodologies and gene panels see supplementary material. Case 10 (presented in Table 4) also had clinical signs of motor delay. ^‡^Cases 4, 8, 9 and 12 have been reported previously.^30^ ID, identifier; CK-MM, creatine kinase-MM; DMD, Duchenne muscular dystrophy; NMD, neuromuscular disease; het, heterozygous; LP, likely pathogenic; P, pathogenic; VUS, variant of uncertain significance; M, male; F, female; EGL, Emory Genetics Laboratory; PEG, Perkin Elmer Genomics; N/A, not applicable; MDA, Muscular Dystrophy Association; NM, neuromuscular; AD, autosomal dominant; AR, autosomal recessive; seq/del & dup, sequencing/deletion and duplication analysis; HIE, hypoxic ischemic encephalopathy.
