# Supplementary material for: Newborn screening for Duchenne muscular dystrophy: A two‐year pilot study
Source: Ann Clin Transl Neurol. 2023 Jun 23;10(8):1383–96. doi: 10.1002/acn3.51829 (PMC10424650; doi:10.1002/acn3.51829)
Supplement: Supplementary file 4 — File S1. [file ACN3-10-1383-s002.docx]

Emory Genetics Laboratory (EGL) methodology and gene panel:

For *DMD* gene sequencing, in solution hybridization of the 79 coding exons, the muscle promoter as well as the region surrounding several known deep intronic pathogenic variants, within the *DMD* gene was performed on the individuals’ genomic DNA. Direct sequencing of the amplified captured regions was performed using next generation short base read sequencing. A minimum of 15x coverage was required to consider a variant as reportable. High quality single nucleotide variants (SNVs) which pass EGL’s quality filters were not confirmed. Reportable SNVs that did not pass the quality filters and all reportable small deletions and duplications were confirmed using bidirectional Sanger sequence analysis. Sequence analysis was validated to detect small deletions of up to 57 bp and small duplications of up to 27 bp in size. Intronic variants greater than 10 nucleotides from exon/intron boundaries were not analyzed and variants greater than three nucleotides from the intron/exon boundary were not reported unless known to be pathogenic. In some cases, due to the complexity of the sequence, not all variants in the flanking intronic sequence could be analyzed.

For expanded neuromuscular disorders sequencing panel, in solution hybridization of the targeted coding exons within the genes tested [Expanded NMD Panel (46 genes) listed below] was performed on the individual’s genomic DNA. The genes on the panel were chosen through evidence-based analysis. Direct sequencing of the amplified capture regions was performed using next generation short base pair read sequencing. A minimum of 15x coverage was required to consider the variant as reportable and any reportable variant not passing the quality filters was confirmed using Sanger sequencing. Exons with inadequate coverage or quality by next generation sequencing were assessed with Sanger sequencing. Sequence analysis was validated to detect small deletions of up to 57 bp and small duplications of up to 27 bp in size. Intronic variants within 10 nucleotides from the exon/intron boundaries were analyzed, unless prohibited by the complexity of the sequence. Additionally select intronic variants beyond 10 nucleotides that have been reported in the literature may have been analyzed. This test included analysis of the SECIS region of the 3’UTR5 of the *SELENON* gene. The test does not detect the retrotransposon insertion in the 3’UTR of the *FKTN* gene common in some Asian populations.

EGL 46 gene sequencing panel:

*ACTA1, AMPD1, ANO5, CAPN3, CAV3, COL6A1, COL6A2, COL6A3, CRPPA, DES, DMD, DYSF, EMD, FKRP, FKTN, GAA, GNE, ITGA7, LAMA2, LARGE1, LMNA, MYOT, NEB, PLEC, PMM2, POMGNT1, POMT1, POMT2, PYGM, RYR1, RYR2, SELENON, SGCA, SGCB, SGCD, SGCE, SGCG, SIL1, TCAP, TNNI2, TNNT1, TPM2, TPM3, TRIM32, TTN, VCP*

For deletion duplication analysis genomic DNA was analyzed using a comparative genomic hybridization (CGH) array custom designed for analyzing the genes on this panel (listed above not including *VCP*). Rarely, probe coverage may have been limited or absent in some exons due to the features of the targeted sequence.

EGL 46 gene deletion/duplication panel:

*ACTA1, AMPD1, ANO5, CAPN3, CAV3, COL6A1, COL6A2, COL6A3, CRPPA, DES, DMD, DYSF, EMD, FKRP, FKTN, GAA, GNE, ITGA7, LAMA2, LARGE1, LMNA, MYOT, NEB, PLEC, PMM2, POMGNT1, POMT1, POMT2, PYGM, RYR1, RYR2, SELENON, SGCA, SGCB, SGCD, SGCE, SGCG, SIL1, TCAP, TNNI2, TNNT1, TPM2, TPM3, TRIM32, TTN, VCP*

Invitae methodology and gene panels:

Genomic DNA obtained from the submitted sample was enriched for targeted regions using a hybridization-based protocol and sequenced using Illumina technology. Unless otherwise indicated, all targeted regions were sequenced with ≥50x depth or were supplemented with additional analysis. Reads were aligned to a reference sequence (GRCh37), and sequence changes were identified and interpreted in the context of a single clinically relevant transcript, indicated below. Enrichment and analysis focused on the coding sequence of the indicated transcripts, 20bp of flanking intronic sequence, and other specific genomic regions demonstrated to be causative of disease at the time of assay design. Promoters, untranslated regions, and other non-coding regions were not otherwise interrogated. For some genes only targeted loci were analyzed (indicated in the table above). Exonic deletions and duplications were called using an in-house algorithm that determines copy number at each target by comparing the read depth for each target in the proband sequence with both mean read-depth and read-depth distribution, obtained from a set of clinical samples. Markers across the X and Y chromosomes were analyzed for quality control purposes and may detect deviations from the expected sex chromosome complement. Such deviations may have been included in the report in accordance with internal guidelines. All clinically significant observations were confirmed by orthogonal technologies, except individually validated variants and variants previously confirmed in a first-degree relative. Confirmation technologies included any of the following: Sanger sequencing, Pacific Biosciences SMRT sequencing, MLPA, MLPA-seq, Array CGH. Array CGH confirmation of NGS CNV calling performed by Invitae Corporation (1400 16th Street, San Francisco, CA 94103, #05D2040778). The following analyses were performed if relevant to the requisition. For *PMS2* exons 12-15, the reference genome was modified to force all sequence reads derived from *PMS2* and the *PMS2CL* pseudogene to align to *PMS2*, and variant calling algorithms were modified to support an expectation of 4 alleles. If a rare SNP or indel variant was identified by this method, both *PMS2* and the *PMS2CL* pseudogene were amplified by long-range PCR and the location of the variant was determined by Pacific Biosciences (PacBio) SMRT sequencing of the relevant exon in both long-range amplicons. If a CNV was identified, MLPA or MLPA-seq was run to confirm the variant. If confirmed, both *PMS2* and *PMS2CL* were amplified by long-range PCR, and the identity of the fixed differences between *PMS2* and *PMS2CL* were sequenced by PacBio from the long-range amplicon to disambiguate the location of the CNV. Technical component of confirmatory sequencing was performed by Invitae Corporation (1400 16th Street, San Francisco, CA 94103, #05D2040778). Technical component of Fibroblast cell-culturing and gDNA extraction from skin punch biopsy was performed by Invitae Corporation (5 Technology Drive, Irvine CA 92618, #05D1052995).

Invitae 109 gene panel:

*ACTA1, AGRN, ALG2, ANO5, ATP2A1, B3GALNT2, B4GAT1, BAG3, BIN1,CACNA1S, CAPN3, CAV3, CCDC78, CFL2, CHAT, CHKB, CHRNA1, CHRNB1, CHRND, CHRNE, CLCN1, CNTN1, COL12A1, COL6A1, COL6A2, COL6A3, COLQ, CPT2, CRYAB, DAG1, DES, DMD, DNAJB6, DNM2, DOK7, DPAGT1, DPM1, DPM2, DPM3, DYSF, EMD, FHL1, FKBP14, FKRP, FKTN, FLNC, GAA, GFPT1, GMPPB, GNE, GYS1, ISPD, ITGA7, KBTBD13, KCNJ2, KLHL40, KLHL41, LAMA2, LAMP2, LARGE1, LDB3, LMNA, LMOD3, MATR3, MEGF10, MTM1, MUSK, MYH2, MYH7, MYL2, MYOT, MYPN, NEB, PLEC, PNPLA2, POMGNT1, POMGNT2, POMK, POMT1, POMT2, PREPL, RAPSN, RXYLT1, RYR1, SCN4A, SELENON, SGCA, SGCB, SGCD, SGCG, SLC5A7, SMN1, SMN2, SQSTM1, STAC3, STIM1, TAZ, TCAP, TIA1,TNNT1, TNPO3, TOR1AIP1, TPM2, TPM3, TRAPPC11, TRIM32, TTN, VCP, VMA21.*

Invitae 122 gene panel:

*ACTA1, AGRN, ALG14, ALG2, ANO5, ATP2A1, B3GALNT2, B4GAT1, BAG3, BIN1, CACNA1S, CAPN3, CAV3, CCDC78, CFL2, CHAT, CHKB, CHRNA1, CHRNB1, CHRND, CHRNE, CLCN1, CNTN1, COL12A1, COL6A1, COL6A2, COL6A3, COLQ, CPT2, CRYAB, DAG1, DES, DMD, DNAJB6, DNM2, DOK7, DPAGT1, DPM1, DPM2, DPM3, DYSF, EMD, FHL1, FKBP14, FKRP, FKTN, FLNC*, GAA, GFPT1, GMPPB, GNE, GYS1, HNRNPA2B1, HNRNPDL, ISPD, ITGA7, KBTBD13, KCNJ2, KLHL40, KLHL41, LAMA2, LAMB2, LAMP2, LARGE1, LDB3, LIMS2, LMNA, LMOD3, LRP4, MATR3, MEGF10, MTM1, MUSK, MYF6, MYH2, MYH7, MYL2, MYOT, MYPN, NEB*, PLEC, PNPLA2, POMGNT1, POMGNT2, POMK, POMT1, POMT2, PREPL, RAPSN, RXYLT1, RYR1, SCN4A, SELENON, SGCA, SGCB, SGCD, SGCG, SLC5A7, SMN1, SMN2, SNAP25, SQSTM1, STAC3, STIM1, SUN1, SUN2, SYNE1, SYNE2, TAZ, TCAP, TIA1, TMEM43, TNNT1, TNPO3, TOR1AIP1, TPM2, TPM3, TRAPPC11, TRIM32, TTN*, VCP, VMA21.*

Invitae 131 gene panel:

*ACTA1, ADSSL1, AGRN, ALG14, ALG2, AMPD1, ANO5, ATP2A1, B3GALNT2, B4GAT1, BAG3, BIN1, CACNA1S, CAPN3*, CASQ1, CAV3, CCDC78, CFL2, CHAT, CHKB, CHRNA1, CHRNB1, CHRND, CHRNE, CLCN1, CNTN1, COL12A1, COL13A1, COL6A1, COL6A2, COL6A3, COLQ, CPT2, CRYAB, DAG1, DES, DMD, DNAJB6, DNM2, DOK7, DPAGT1, DPM1, DPM2, DPM3, DYSF, EMD, FHL1, FKBP14, FKRP, FKTN, FLNC*, GAA, GFPT1, GMPPB, GNE, GOSR2, GYG1, GYS1, HACD1, HNRNPA2B1, HNRNPDL, ISCU, ISPD, ITGA7, KBTBD13, KCNJ2, KLHL40, KLHL41, LAMA2, LAMP2, LARGE1, LDB3, LMNA, LMOD3, MAP3K20, MATR3, MEGF10, MICU1, MTM1, MUSK, MYH2, MYH7, MYL2, MYO18B, MYOT, MYPN, NEB*, ORAI1, PLEC, PNPLA2, POMGNT1, POMGNT2, POMK, POMT1, POMT2, PREPL, PYROXD1, RAPSN, RXYLT1, RYR1, SCN4A, SELENON, SGCA, SGCB, SGCD, SGCG, SLC18A3, SLC5A7, SMCHD1, SMN1, SMN2, SPEG, SQSTM1, STAC3, STIM1, SYT2, TAZ, TCAP, TIA1, TK2, TNNT1, TNNT3, TNPO3, TOR1AIP1, TPM2, TPM3*, TRAPPC11, TRIM32, TTN*, VAMP1, VCP, VMA21.*

Invitae 143 gene panel:

*ACTA1, ADSSL1, AGRN, ALG14, ALG2, AMPD1, ANO5, ATP2A1, B3GALNT2, B4GAT1, BAG3, BIN1, CACNA1S, CAPN3*, CASQ1, CAV3, CCDC78, CFL2, CHAT, CHKB, CHRNA1, CHRNB1, CHRND, CHRNE, CLCN1, CNTN1, COL12A1, COL13A1, COL6A1, COL6A2, COL6A3, COLQ, CPT2, CRYAB, DAG1, DES, DMD, DNAJB6, DNM2, DOK7, DPAGT1, DPM1, DPM2, DPM3, DYSF, EMD, FHL1, FKBP14, FKRP, FKTN, FLNC*, GAA, GFPT1, GMPPB, GNE, GOSR2, GYG1, GYS1, HACD1, HNRNPA2B1, HNRNPDL, ISCU, ISPD, ITGA7, KBTBD13, KCNJ2, KLHL40, KLHL41, KLHL9, LAMA2, LAMB2, LAMP2, LARGE1, LDB3, LIMS2, LMNA, LMOD3, LRP4, MAP3K20, MATR3, MEGF10, MICU1, MTM1, MTMR14, MUSK, MYH2, MYH7, MYL2, MYO18B, MYOT, MYPN, NEB*, ORAI1, PLEC, PNPLA2, POMGNT1, POMGNT2, POMK, POMT1, POMT2, PREPL, PYROXD1, RAPSN, RXYLT1, RYR1, SCN4A, SELENON, SGCA, SGCB, SGCD, SGCG, SLC18A3, SLC5A7, SMCHD1, SMN1, SMN2, SNAP25, SPEG, SQSTM1, STAC3, STIM1, SUN1, SUN2, SYNE1, SYNE2, SYT2, TAZ, TCAP, TIA1, TK2, TMEM43, TNNT1, TNNT3, TNPO3, TOR1AIP1, TPM2, TPM3*, TRAPPC11, TRIM32, TTN*, VAMP1, VCP, VMA21.*

Invitae 230 gene panel:

*ABHD5, ACAD9, ACADM, ACADVL, ACTA1, ADSSL1, AGK, AGL, AGRN, AHCY, ALDOA, ALG14, ALG2, AMACR, AMPD1, ANO5, ATP2A1, ATP7B, B3GALNT2, B4GAT1, BAG3, BIN1, C1QBP, CACNA1S, CAPN3, CASQ1, CAV3, CCDC78, CFL2, CHAT, CHKB, CHRNA1, CHRNB1, CHRND, CHRNE, CLCN1, CNTN1, COL12A1, COL13A1, COL6A1, COL6A2, COL6A3, COLQ, COQ2, COQ4, COQ7, COQ8A, COQ9, COX15, COX20, COX6B1, CPT1A, CPT2, CRYAB, CTDP1, DAG1, DDC, DES, DGUOK, DMD, DNA2, DNAJB6, DNM2, DOK7, DPAGT1, DPM1, DPM2, DPM3, DYSF, EMD, ENO3, ETFA, ETFB, ETFDH, FBXL4, FDX2, FHL1, FKBP14, FKRP, FKTN, FLAD1, FLNC, GAA, GATM, GBE1, GFER, GFPT1, GMPPB, GNE, GOSR2, GYG1, GYS1, HACD1, HADH, HADHA, HADHB, HMBS, HNRNPA2B1, HNRNPDL, ISCU, ISPD, ITGA7, KBTBD13, KCNJ2, KLHL40, KLHL41, LAMA2, LAMP2, LARGE1, LDB3, LDHA, LMNA, LMOD3, LPIN1, MAN2B1, MAP3K20, MATR3, MEGF10, MGME1, MICU1, MPV17, MTM1, MUSK, MYH2, MYH3, MYH7, MYL2, MYO18B, MYOT, MYPN, NEB, OPA1, OPA3, ORAI1, PDSS1, PDSS2, FKM, PGAM2, PGK1, PGM1, PHKA1, PHKB, PLEC, PNPLA2, PNPLA8, POGLUT1, POLG, POLG2, POMGNT1, POMGNT2, POMK, POMT1, POMT2, PREPL, PUS1, PYGM, PYROXD1, RAPSN, RBCK1, RNASEH1, RRM2B, RXYLT1, RYR1, SCN4A, SDHA, SELENON, SGCA, SGCB, SGCD, SGCG, SIL1, SLC16A1, SLC18A3, SLC22A5, SLC25A20, SLC25A3, SLC25A4, SLC25A42, SLC5A7, SMCHD1, SMN1, SMN2, SMPX, SPEG, SQSTM1, STAC3, STIM1, SUCLA2, SUCLG1, SYT2, TANGO2, TAZ, TCAP, TIA1, TK2, TNNT1, TNPO3, TOR1AIP1, TPM2, TPM3, TRAPPC11, TRIM32, TRMT5, TSFM, TTN, TWNK, TYMP, VAMP1, VCP, VMA21, YARS2*. Add-on Preliminary-evidence Genes for Neuromuscular Disorders (19 genes):*ATP1A2, ATP5D, ATP5E, CHCHD10, KLHL9, LAMB2, LIMS2, LRP4, MCM3AP, MTMR14, SLC25A32, SNAP25, SUN1, SUN2, SYNE1, SYNE2, TMEM43, TNNT3, TOP3A*.

Perkin Elmer Genomics (PEG) methodology and gene panels:

The genes included in this assay were selected by the ordering provider. Sequencing was performed on genomic DNA using an Agilent targeted sequence capture method to enrich for the exome. Direct sequencing of the amplified captured regions was performed using 2x150bp reads on Illumina next generation sequencing (NGS) systems. Alignment to the human reference genome (hg19) was performed and annotated variants were identified in the targeted region.

Variants reviewed had a minimum coverage of 8x and an alternate allele frequency of 20% or higher. Indel and single nucleotide variants (SNVs) may have been confirmed by Sanger sequence analysis before reporting at director discretion. This assay could not detect variants in regions of the exome that were not covered, such as deep intronic, promoter and enhancer regions, areas containing large numbers of tandem repeats, and variants in mitochondrial DNA. Copy number variation (CNV) analysis was designed to detect deletions and duplications of three exons or more; in some instances, due to the size of the exons or other factors, not all CNVs may have been analyzed. CNV analysis will not detect tandem repeats, balanced alterations (reciprocal translocations, Robertsonian translocations, inversions, and balanced insertions), methylation abnormalities, triploidy, and genomic imbalances in segmentally duplicated regions. This assay was not designed to detect mosaicism; possible cases of mosaicism may have been investigated at the discretion of the laboratory director. Primary data analysis was performed using Illumina bcl2fastq converter v2.19. Secondary analysis was performed using Illumina DRAGEN Bio-IT Platform v.3.4.12. Tertiary data analysis was performed using SnpEff v5.0 and PerkinElmer’s internal ODIN v.1.01 software. CNV and absence of heterozygosity were assessed using BioDiscovery’s NxClinical v5.1 software.

Initially the *DMD* gene sequence was analyzed. If no pathogenic/likely pathogenic (P/LP) variants were detected, results of the 46 gene panel below were analyzed and if no P/LP variants were detected, results of the 90 gene panel below were analyzed.

PEG gene panel (46 genes):

*ACTA1, AMPD1, ANO5, CAPN3, CAV3, COL6A1, COL6A2, COL6A3, CRPPA, DES, DMD, DYSF, EMD, FKRP, FKTN, GAA, GNE, ITGA7, LAMA2, LARGE1, LMNA, MYOT, NEB, PLEC, PMM2, POMGNT1, POMT1, POMT2, PYGM, RYR1, RYR2, SELENON, SGCA, SGCB, SGCD, SGCE, SGCG, SIL1, TCAP, TNNI2, TNNT1, TPM2, TPM3, TRIM32, TTN, VCP.*

PEG gene panel (90 genes):

*AGRN, ALG14, ALG2, ASAH1, ATP2A1, B3GALNT2, B4GAT1, BAG3, BICD2, BIN,1 CACNA1S, CCDC78, CFL2, CHAT, CHKB, CHRNA1, CHRNB1, CHRND, CHRNE, CLCN1, CNTN1, COL12A1, COLQ, CPT2, CRYAB, DAG1, DNAJB6, DNM2, DOK7, DPAGT1, DPM1, DPM2, DPM3, DYNC1H1, FHL1, FKBP14, FLNC, GFPT1, GMPPB, HNRNPA2B1, HNRNPDL, IGHMBP2, KBTBD13, KCNJ2, KLHL40, KLHL41, LAMB2, LAMP2, LDB3, LIMS2, LMOD3, LRP4, MATR3, MEGF10, MTM1, MUSK, MYF6, MYH2, MYH7, MYL2, MYPN, PHKA1, PLEKHG5, PMP22, PNPLA2, POMGNT2, POMK, PREPL, RAPSN, RXYLT1, SCN4A, SMCHD1, SNAP25, SQSTM1, STAC3, STIM1, SUN1, SUN2, SYNE1, SYNE2, TAFAZZIN, TIA1, TMEM43, TNPO3, TOR1AIP1, TRAPPC11, TRPV4, UBA1, VMA21, VRK1.*
